# Validation of a childhood eating disorder outcome scale

**DOI:** 10.1186/s13030-019-0162-3

**Published:** 2019-09-11

**Authors:** Shinichiro Nagamitsu, Yoshimitsu Fukai, So Uchida, Michiko Matsuoka, Toshiyuki Iguchi, Ayumi Okada, Ryoichi Sakuta, Takeshi Inoue, Ryoko Otani, Shinji Kitayama, Kenshi Koyanagi, Yuichi Suzuki, Yuki Suzuki, Yoshino Sumi, Shizuo Takamiya, Chikako Fujii, Yasuko Tsurumaru, Ryuta Ishii, Tatsuyuki Kakuma, Yushiro Yamashita

**Affiliations:** 1 Department of Pediatrics and Child Health, Kurume University School of Medicine, 67 Asahi-machi, Kurume, Fukuoka, 830-0011 Japan; 2 Psychosomatic Medicine, Tokyo Metropolitan Children’s Medical Center, Tokyo, Japan; 3 Department of Pediatrics, Tachikawa Hospital, Tachikawa, Japan; 4 Department of Neuropsychiatry, Kurume University School of Medicine, Kurume, Japan; 5 Department of Pediatrics Hoshigaoka Maternity Hospital, Nagoya, Japan; 6 Department of Pediatrics, Okayama University Graduate School of Medicine, Dentistry and Pharmaceutical Sciences, Okayama, Japan; 7 Child Development and Psychosomatic Medicine Center, Dokkyo Medical University Saitama Medical Center, Saitama, Japan; 8 Himeji City Center for the Disabled, Himeji, Japan; 9 Nagasaki Prefectural Center of Medicine and Welfare for Children, Nagasaki, Japan; 10 Department of Pediatrics, Fukushima Medical University School of Medicine, Fukushima, Japan; 11 Department of Pediatrics, National Hospital Organization Mie National Hospital, Mie, Japan; 12 Department of Pediatrics, School of Medicine, Sapporo Medical University, Sapporo, Japan; 13 Mental and Developmental Clinic for Children “ELM TREE”, Sapporo, Japan; 14 Psychiatry Department, Kobe City Nishi-Kobe Medical Center, Kobe, Japan; 15 Takamiya Medical Clinic, Akashi, Japan; 16 Department of Pediatrics, Okayama University Hospital, Okayama, Japan; 17 Biostatistics Center, Kurume University, Kurume, Japan

**Keywords:** Eating disorders, Anorexia nervosa, Outcome, Children

## Abstract

We developed and validated a childhood eating disorder outcome scale based on outcomes associated with body mass index standard deviation score (BMI-SDS). This prospective observational study included 131 children with eating disorders (aged 5–15 years). Participants’ outcomes scales were completed at the first visit and at 1, 3, 6, and 12 months. The scale evaluated 12 outcomes: body weight change (BW), eating attitude (EA), fear of being fat (FF), body image distortion (BD), menstruation (ME), perceived physical condition (PC), attending school (AS), disease recognition by school (RS), family function (FA), disease recognition by parent (RP), social adaptation (SA), and relationships with friends (RF). Responses to all items were on a four-point Likert scale. Exploratory factor analysis was used to determine the number of factors based on the 12 outcomes. The relation between outcome scale scores and BMI-SDS over the 12-month follow-up period was analyzed. Two types of factors were extracted: disease-specific factors (EA, FF, BD) and biopsychosocial factors (BW, PC, AS, FA, SA, RF). Three items (ME, RS, RP) were excluded because they showed no significant loading effect. There was a significant negative correlation between the outcome scale and BMI-SDS, and changes in outcome scale scores from baseline to 12 months were significantly associated with improvement in BMI-SDS. We developed a childhood eating disorder outcome scale characterized by disease-specific and biopsychosocial factors. Biopsychosocial management combined with a therapeutic approach for disease-specific symptoms may support body weight recovery for children with eating disorders.

## Introduction

Childhood eating disorders are serious psychiatric illnesses that confer substantial morbidity and mortality and manifest as disturbances in eating habits, excessive preoccupation with weight, restricted caloric intake, and body image distortion. The prevalence of childhood eating disorder is 1% in adolescents, with one-third of these adolescents classified as avoidant/restrictive food intake disorder (ARFID) and more than half as anorexia nervosa (AN) [1–4]. Various short-term and longitudinal outcome studies have reported 40–88% complete remission, 34–72% recurrence, and 12–24% undesirable outcomes) [5–11]. However, the results varied depending on the definition of outcome used in the study.

The Morgan-Russell Outcome Assessment Schedule was developed to identify outcome predictors using data from clinical observation, premorbid personality, and the patient’s family background at the time of first assessment [12]. It covers nutritional status, menstrual function, mental state, sexual adjustment, and socioeconomic status; however, those scales are not standardized and each rating scale is relatively subjective. Furthermore, some scales are not specific to children, as they cover sexual relationships, establishment of economic independence from the family, and occupational records. Moreover, the Morgan-Russell Outcome scale does not include core symptoms of eating disorders, such as body image distortion and fear of being fat. The Morgan-Russell Outcome Scale is widely used for eating disorders in adulthood [9, 13, 14]; however, many studies used body weight and the presence of menstruation to evaluate prognosis. Similarly, the Diagnostic and Statistical Manual of Mental Disorders, fifth Edition (DSM-5) definition of eating disorders specifies disease severity using body mass index (BMI); for example, moderate (BMI 16–16.99 kg/m^2^), severe (BMI 15–15.99 kg/m^2^), and extreme (BMI < 15 kg/m^2^) [15]. However, absolute BMI values are not recommended as a measure for underweight in adolescence, as development-related variations may bias results [16, 17]. In DSM-5, an age- and gender-adjusted BMI percentile of 5 is suggested as a numeric cut-off for low weight criterion in AN in children and adolescents, however, the evidence supporting this cut-off for underweight is lacking [18]. As AN cases with extreme low weight are in a BMI percentile near zero, it is difficult to distinguish their severity of weight loss. Therefore, BMI standard deviation scores (BMI-SDS), which are also age- and sex-standardized, may represent a more accurate measure [16, 17]. Furthermore, recent increments in early onset of childhood AN may affect onset of menarche, meaning the presence or absence of menstruation may not be a suitable outcome measurement in child cases. Therefore, an eating disorder outcome scale that is specific for children’s developmental physical condition is needed.

In addition to consideration of physical developmental condition, psychosocial factors (e.g., family function, relationships with friends, and school adaptation) are often considered for recovery in childhood eating disorder [19, 20]. Several studies have argued the importance of family functioning in the recovery process for children with AN [21, 22]. There is an association between an adolescent’s positive perception of family functioning and better outcomes. However, family distress and dysfunction, such as parental conflict or feelings of depression, predict poorer outcomes [23–27]. Relationships with friends and school achievement also have a significant impact on recovery [28–30]. Westwood et al. [30] reported that social difficulties may play a role in both the development and maintenance of AN, especially where there is isolation from the friendship experience. Furthermore, positive friendship qualities, such as good communication, trust, and peer acceptance were not associated with eating disorder symptoms [28, 30]. A national cohort study showed high achievement in school was associated with an increased risk for eating disorders [29], although the reason for this association remained unknown. However, for example, if a child with a perfectionist tendency has experienced less than perfect school achievement, this characteristic may prohibit their recovery from AN. This indicates that a childhood eating disorder outcome scale should include psychosocial assessment factors in addition to physical assessment, as both psychosocial and physical improvements are needed for complete recovery.

This study aimed to develop and validate a childhood eating disorder outcome scale that reflects both physical and psychosocial assessment, in addition to evaluating core eating disorder symptoms. We conducted a prospective observational study involving 131 children with eating disorders. To ensure the outcome scale was reliable, we used BMI-SDS to reflect weight changes, performed factor analysis to eliminate low loading items, and developed distinct definitions for each item.

## Methods

### Study organization

The Japanese Pediatric Eating Disorders Outcome: a Prospective Multicenter Cohort Study (J-PED study) group was established in 2014 to develop a childhood eating disorder outcome scale. Eleven regional medical institutions covering northern to southern Japan participated in this study. Participating medical institutions employed pediatricians who were engaged in managing treatment for children with eating disorders. Members of the J-PED study team discussed several possible items for the outcome scale, and decided on the definitions of the clinical level for each outcome. All J-PED members were also trained by a child psychiatrist on how to use the Mini-International Neuropsychiatric Interview (MINI) [31] to assess comorbid psychiatric disorders.

### Study protocol

#### Enrollment

The purpose of this study was to develop a childhood eating disorder outcome scale associated with BMI-SDS. Inclusion criteria were: aged under 16 years, having abnormal eating habits and unaccountable weight change. The J-PED members ruled out medical causes for the eating habits and the weight change, and checked for any related complications. After patients had physical examination and laboratory tests, their eating habits were assessed by the Japanese version of the ChEAT26 [32]. If the J-PED members suspected that patients have some type of eating disorder, they used the diagnostic criteria for eating disorders in DSM-IV, Great Ormond Street Criteria or DSM-5) [15, 33]. They also used MINI to assess any comorbid psychiatric disorders. Untreated first-visit patients and patients who were referred from other hospitals were included. Patients who did not provide informed consent to participate in this study were excluded. The enrollment period was from 2014 April to 2016 March (2 years).

#### Participants

Patients with eating disorders who visited one of the J-PED study group medical institutions for treatment received an explanation about the study protocol. Those who understood the purpose of this study and provided signed informed consent or assent were eligible to participate in this study. In total, 131 children with eating disorders were enrolled between 2014 and 2016; 89 were diagnosed as AN, and 38 as ARFID. The remaining four were diagnosed with functional dysphagia. There were no cases with bulimia nervosa. The mean (standard deviation [SD]) onset age was 12.5 years (1.9 years). The number of inpatient and outpatient cases were 82 and 49, respectively. The mean and standard deviation of the duration between possible disease onset and first visit was 262 days and 245 days, respectively.

#### Candidate items for the outcome scale

We referred to several papers to select optimal candidate items for the outcome scale [5, 34–37], and extracted many items that manifest a multidimensional measure of eating disorder pathology. After discussion among the J-PED members, we classified these items into similar categories and selected 12 outcomes: 1) body weight change (BW), 2) eating attitudes (EA), 3) fear of being fat (FF), 4) body image distortion (BD), 5) menstruation (ME), 6) perception of physical condition (PC), 7) family function (FA), 8) disease recognition by parent (RP), 9) disease recognition by school (RS), 10) attending school (AS), 11) relationships with friends (RF), and 12) social adaption (SA). There were four possible responses for each outcome (e.g., 0 = no problem, 1 = unclear, 2 = moderate problem, and 3 = major problem). After discussion among the J-PED members, detailed definitions for each point on all outcomes were developed. For example, the BW outcome (evaluated at each visit) was categorized into four levels of BMI-SDS change: >2SD decrease (scored as 3), 1SD–2SD decrease (scored as 2), between 1SD increase and < 1SD decrease (scored as 1), and a > 1SD increase (scored as 0). The FF item was based on three parameters: intense fear of becoming fat, preoccupation with thinness, and excessive exercise. If a patient met all three parameters, the score was recorded as 3. If a patient had none, one, or two of the three parameters, the score was 0, 1, or 2, respectively. A patient’s score for the outcome therefore depended on how many parameters that patient met. PC were assessed in same manner. The scale definition of “body image distortion (BD)” was derived from criteria C for AN in the DSM-5. Criteria C specifies: disturbance in the way in which one’s body weight or shape is experienced, undue influence of body weight or shape on self-evaluation, or persistent lack of recognition of the seriousness of current low body weight. We applied the second and third points of criteria C without any changes as parameters in this study; however, we represented the first point as “preoccupation with body image.” ME was assessed by regularity of menstruation and AS by the frequency of attending school. Definitions of the EA, FA, RP, RS, RE, and SA scores are described using examples (Table 1). The total score for the childhood eating disorder outcome scale ranged from 0 to 36, with a score of 36 indicating the worst condition. Before this study started, physicians self-trained in using the outcome scale by rating imaginary cases with eating disorders. After rating, they discussed the scores in each subscale that were suitable for the imaginary cases as a group. This aimed to preserve inter-rater reliability across institutions and within the whole study group.

**Table 1:** Outcome scale candidate items and definition of scores

1. Body weight change (BW)	
score 0 increase 1SD	
score 1 change ±1SD	
score 2 decrease >1SD − 2SD	
score 3 decrease >2SD	
2. Eating attitude (EA)	
score 0 no problem	
score 1 unclear	
score 2 moderate problem	
score 3 major problem	
3. Fear of being fat (FF)	
Parameters: 1) intense fear of becoming fat	
2) preoccupation with thinness	
3) excessive exercise	
score 0 none of the parameters	
score 1 any one parameter	
score 2 any two parameters	
score 3 all of the parameters	
4. Body image distortion (BD)	
Parameters: 1) preoccupation with body image	
2) undue influence of body weight or shape on self-evaluation	
3) persistent lack of recognition of seriousness	
score 0 none of the parameters	
score 1 any one parameter	
score 2 any two parameters	
score 3 all of the parameters	
5. Menstruation (ME)	
score 0 regular menstruation	
score 1 unclear	
score 2 irregular menstruation	
score 3 no menstruation	
6. Perceived physical condition (PC)	
Parameters: 1) no feeling of being tired	
2) no feeling of being hungry	
3) no feeling of fullness	
score 0 none of the parameters	
score 1 any one parameter	
score 2 any two parameters	
score 3 all of the parameters	
7. Attending school (AS)	
score 0 going school every day	
score 1 going school a few times/week	
score 2 can go to substitutional school	
score 3 cannot go to any school	
8. Disease recognition by school (RS)	
score 0 positively cooperative	
score 1 slightly cooperative	
score 2 unconcerned	
score 3 critical or refusal	
9. Family function (FA)	
score 0 relationship always good	
score 1 sometimes good, sometimes bad	
score 2 tension at home	
score 3 impossible to be involved	
10. Disease recognition by parent (RP)	
score 0 positively cooperative	
score 1 slightly cooperative	
score 2 unconcerned	
score 3 critical or refusal	
11. Social adaptation (SA)	
score 0 assertive and cooperative	
score 1 unclear	
score 2 in conflict	
score 3 isolated	
12. Relationships with friends (RF)	
score 0 having friends that are trusted	
score 1 having friends to talk with	
score 2 no friends, but not isolated	
score 3 no friends and isolated	

#### Assessment schedule and scales

Participating patients were assessed using the childhood eating disorder outcome scale at their first visit, and at 1, 3, 6, and 12 months after the first visit. Comorbidities (psychiatric disorders and other complications) were assessed at the first visit. In addition, the Japanese version of the ChEAT26 [32] was administered and BMI-SDS calculated at each assessment. In each institution, a single rater performed the evaluations at baseline and at 1, 3, 6, and 12 months.

#### Treatment

Participating patients were treated at either the inpatient or outpatient unit, according to the Childhood Eating Disorder Treatment Protocol published by Japanese Society of Psychosomatic Pediatrics [38]. This guideline includes total parental nutrition therapy, enteral feeding through a nasogastric tube, psychotherapy, behavioral therapy, and medications. A combination of these therapies was selected in each case.

#### Statistical analysis

To verify the validity of the childhood eating disorder outcome scale, we performed a factor analysis for the scale and correlation analysis between the modified scale and BMI-SDS in 131 children with eating disorders at the first visit. We performed correlation analysis between the changes of the scale scores and BMI-SDS from the first visit to the 12 month follow-up visit.

Exploratory factor analysis with varimax rotation was performed to determine the number of factors. Eigenvalues and clinical consideration of factor structures were used to decide the number of factors to be retained. After the number of factors was determined, confirmatory factor analysis was performed to obtain factor loadings. The relation between the outcome scale scores and BMI-SDS at first visit and at 12 months was analyzed with Pearson’s correlation coefficients.

## Results

### Rate of patient follow up and comorbid disorders

**Fig. 1:**
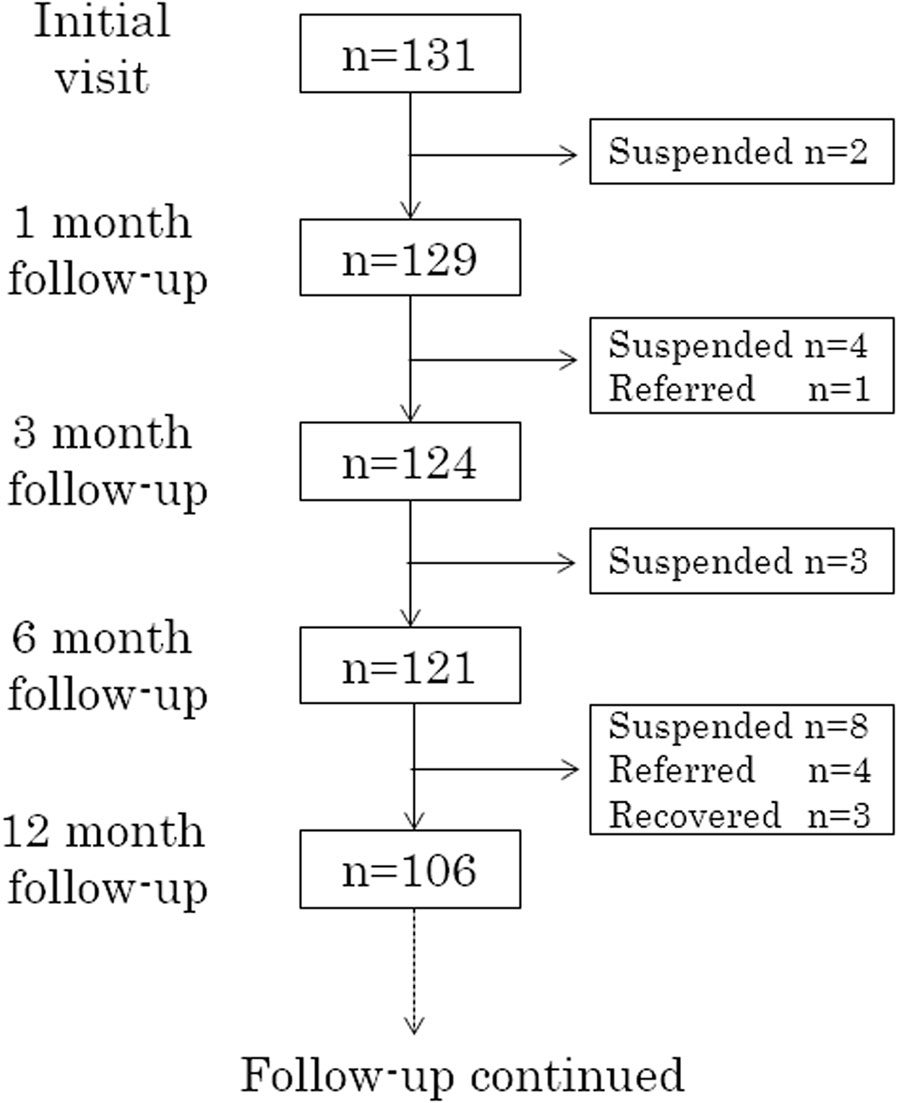
Flow diagram for this study

Before the 12-month follow-up visit, 17 patients stopped visiting their medical institution. The timing of the participants who dropped out is described in Fig. 1. All dropped out before recovering from their eating disorder symptoms. Five patients were referred to a psychiatric clinic because of suicidal behaviors, major depression, or other behavioral problems: Three recovered before the 12-month follow-up visit.

### Patient characteristics

The characteristics of 131 participating children (10 boys) are summarized in Table 2. In total, 89 were diagnosed with AN (86 had restricting type and the remaining three had binge eating/purging type), 38 with ARFID, and four with another form of eating disorder. Of the 10 boys with eating disorders, three had AN and seven had ARFID. Age ranged from 5.5–15.5 years, with a mean age of 12.5 years. Twenty-two girls were younger than 12 years; of these, 12 had AN, nine had ARFID, and one had functional dysphagia. Overall, four children had normal weight loss, five had moderate weight loss, 13 had severe weight loss, and 105 had extreme weight loss; 89% of the children with AN and 82% with ARFID showed extreme weight loss. Forty-six children were diagnosed with psychiatric comorbidity, including anxiety disorder (*n* = 11), suicidal behaviors (*n* = 9), obsessive compulsive disorder (*n* = 7), autistic spectrum disorders (*n* = 8), major depression (*n* = 5), and others (*n* = 6). Fourteen patients had a serious physical illness, such as superior mesenteric artery syndrome or severe infection. One child had a low potassium level (less than 3 mEq/L), 13 had hypotension (systolic blood pressure < 80 mmHg), and 69 had bradycardia (heart rate < 60/min). Over the study period, the mean BMI-SDS changed from − 3.5 to − 1.6, and the mean childhood eating outcome score from 13.3 to 6.2. Among the 46 children with psychiatric comorbidity, data for the first visit showed the mean BMI-SDS was − 3.2 and the mean childhood eating outcome score was 17.0. At the 12-month follow-up, the mean BMI-SDS was − 0.8 and mean childhood eating outcome score was 8.3. Total parental nutrition was reported for 14 children and enteral feeding through a nasogastric tube was reported for seven children. Forty-three children received counseling, 15 received psychotherapy and eight received cognitive behavioral therapy. Eighteen children were taking psychotropic medication, including risperidone (*n* = 6), aripiprazole (*n* = 4), sulpiride (*n* = 4), fluvoxamine (*n* = 2), and others.

**Table 2:** Patients’ characteristics

	Total cases	AN	ARFID
At the first visit			
N (M:F ratio)	131 (10:121)	89 (3:86)	38 (7:31)
Mean age, y (range)	12.5 (5.5–15.5)	12.9 (9.1–15.5)	11.6 (5.5–14.8)
Mean BMI-SDS (range)	−3.5 (−8.7–0.1)	−3.7 (−7.8–0.1)	−3.3 (− 8.7−−1.1)
Mean outcome score (range)	13.3 (4–23)	14.6 (6–23)	10.4 (5–18)
Mean ChEAT26 (SD)	21.4 (15.0)	25.6 (15.4)	11.6 (8.6)
At the 12-month follow-up			
Mean BMI-SDS (range)	−1.6 (−7.7–1.5)	−1.5 (− 7.7–1.5)	− 1.6 (−6.4–0.1)
Mean outcome score (range)	6.2 (0–21)	6.8 (0–21)	4.4 (0–14)
Mean ChEAT26 (SD)	12.3 (12.3)	14.4 (13.3)	7.8 (8.9)

*AN* anorexia nervosa, *ARFID* avoidant/restrictive food intake disorder, *y* years, *BMI-SDS* body mass index-standard deviation score, *ChEAT26* children’s version of the Eating Attitude Test-26 items, *SD* standard deviation

### Factor analysis of the childhood eating disorder outcome scale

Initial exploratory factor analysis was performed to extract important factors. A two-factor solution explained 39.1% of the total variance and showed appropriate eigenvalues and clinically meaningful factor structures. Based on the results of this analysis, we conducted confirmatory factor analysis (Tables 3 and 4). One factor included three outcomes (EA, FF, and BD) and the other included six outcomes (BW, PC, AS, FA, SA, RF). The first factor reflected core eating disorder symptoms (preoccupation with being thin, a fear of getting fat, and eating attitude), and was called the “disease-specific factor.” The second factor comprised six items covering physical and psychological domains, and was labeled the “biopsychosocial factor.” The outcomes FF and BD of the disease-specific factor showed higher loading effects (Table 4). Three outcomes (ME, RS, RP) were excluded because of low loading.

**Table 3:**
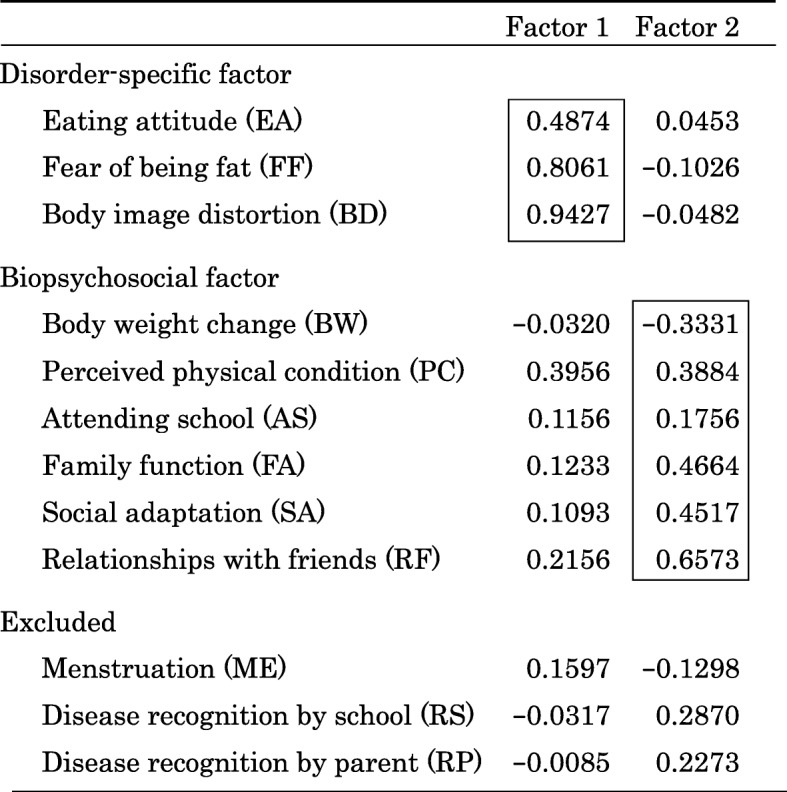
Factor analysis of the childhood eating disorder outcome scale

**Table 4:** Confirmatory factor analysis

Score item	Coef.	Std. Err	Z	*p*-value	95%	CI	Cronbach’s alphas
Disorder-specific factor							
Eating attitude (EA)	0.3114	0.5910	5.27	0.000	0.1956	0.4273	0.6310
Fear of being fat (FF)	0.9574	0.1028	9.32	0.000	0.7560	1.1589	0.6087
Body image distortion (BD)	1.0258	0.9244	11.10	0.000	0.8445	1.2069	0.5919
Biopsychosocial factor							
Body weight change (BW)	−0.2978	0.0960	−3.10	0.002	−0.4860	−0.1097	0.6771
Perceived physical condition (PC)	0.4353	0.0956	4.55	0.000	0.2479	0.6226	0.6064
Attending school (AS)	0.3217	0.1493	2.15	0.031	0.0290	0.6143	0.6667
Family function (FA)	0.3260	0.0785	4.15	0.000	0.1721	0.4799	0.6451
Social adaptation (SA)	0.4571	0.1000	4.55	0.000	0.2601	0.6541	0.6340
Relationships with friends (RF)	0.7279	0.1062	6.86	0.000	0.5198	0.9360	0.5995

*CI* confidence interval

### Correlation between the childhood eating disorder outcome scale and BMI-SDS at the first visit

**Fig. 2:**
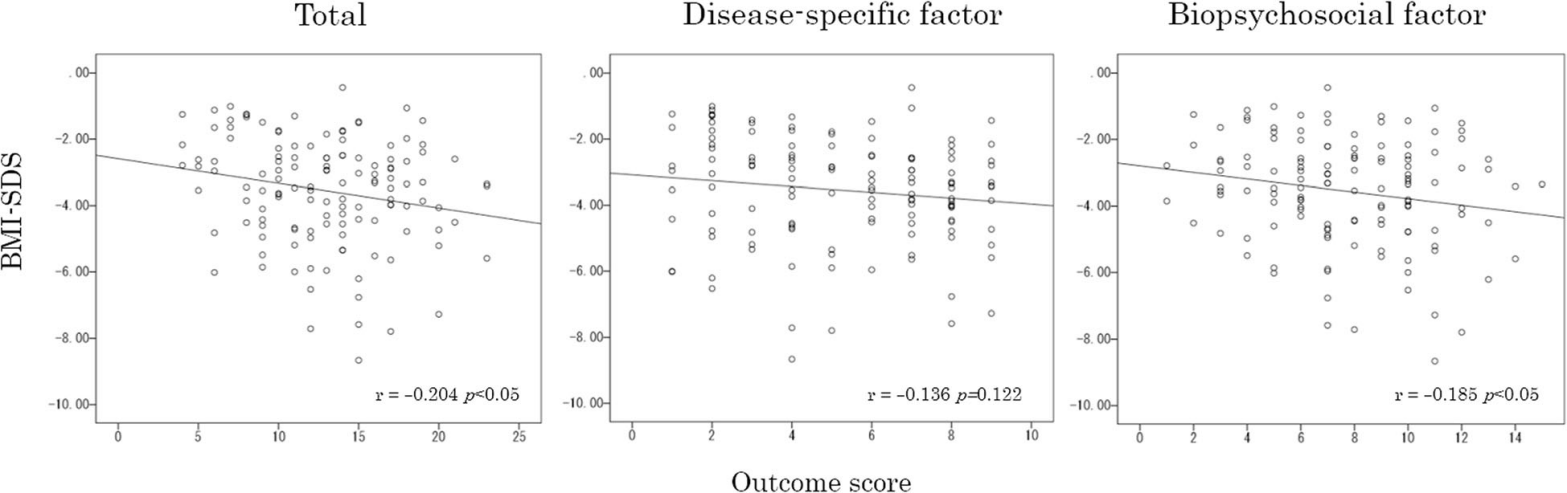
Correlation analysis between each outcome score and BMI-SDS at first visit. BMI-SDS, body mass index standard deviation score

There was a significant negative correlation between the childhood eating disorder outcome scale total score and BMI-SDS at the first visit (*r* = − 0.204, *p* < 0.05) (Fig. 2, left). We further divided the correlation analysis by the disease-specific and biopsychosocial factors) (Fig. 2, middle and right). The sum of the three outcome scores for the disease specific factor showed no correlation with BMI-SDS (Fig. 2, middle); however, the sum of the six outcome scores for the biopsychosocial factor showed a significant negative correlation with the BMI-SDS (Fig. 2, right).

### Correlation between the changes in the childhood eating disorder outcome scale scores and BMI-SDS from the first visit to 12 months

**Fig. 3:**
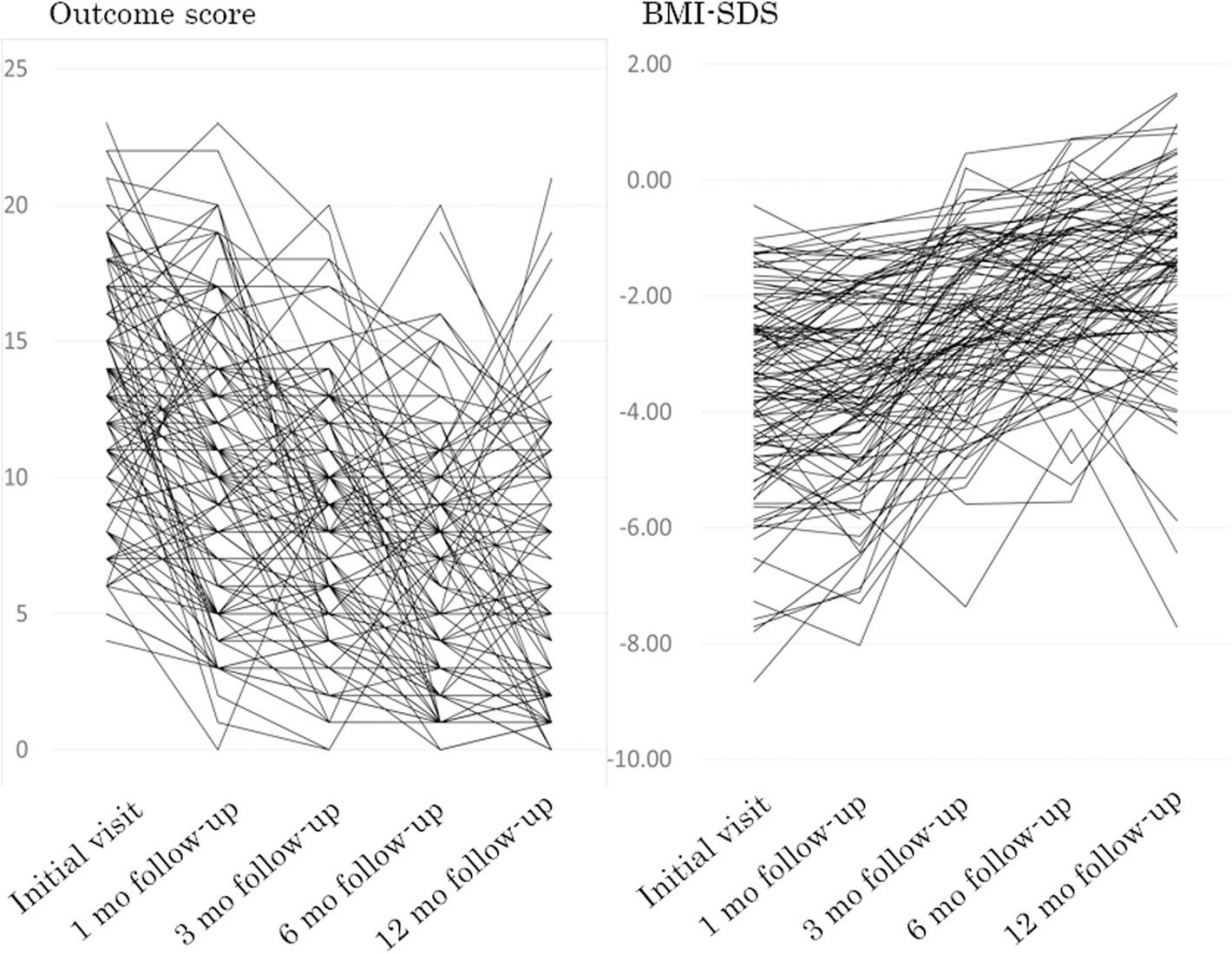
Consecutive outcome scale and BMI-SDS for 131 patients during the study. BMI-SDS, body mass index standard deviation score; mo, month/months

**Fig. 4:**
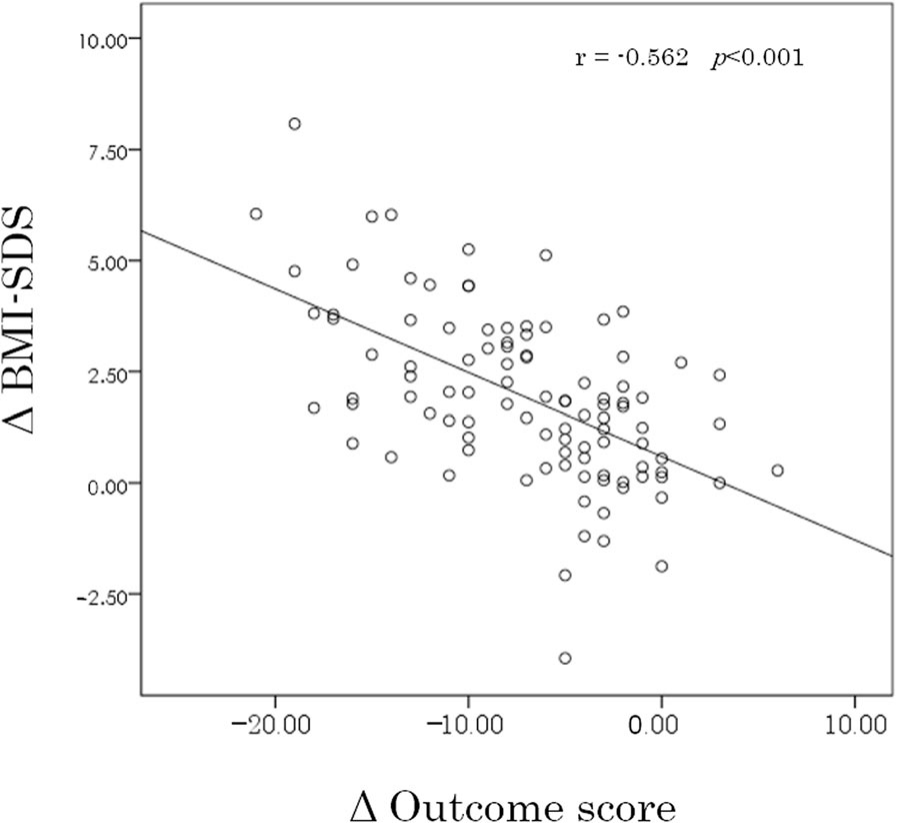
Correlation between changes in outcome score and BMI-SDS from the first visit to the 12-month follow-up evaluation. MI-SDS, body mass index standard deviation score; Δ, Changes of outcome score or BMI-SDS from the initial visit to the 12-month follow-up

Figure 3 describes consecutive outcome scale and BMI-SDS data from the first visit to the 12-month follow-up visit. The changes in each outcome score and BMI-SDS were calculated by subtracting each score at the 12-month follow-up from those at the first visit. The mean and range of changes of the scale score and BMI-SDS were − 7.0 (range − 21 to 6) and 1.9 (range 8.1 to − 4.0), respectively. There was a significant negative correlation between change in the outcome scale score and change in BMI-SDS (*r* = − 0.562, *p* < 0.001) (Fig. 4).

## Discussion

Childhood eating disorder outcome studies have received much interest from child psychiatrists and pediatricians, especially as some childhood cases have revealed poor disease outcomes, with transition to an adult form of the eating disorder [5–7]; however, others have shown a good prognosis [8, 9, 11]. Psychiatrists and pediatricians are interested in the factors that are significant for better prognosis. Therefore, we developed a childhood eating disorder outcome scale that was verified by factor analysis and consecutive application over a 1-year period. Factor analysis revealed a two-factor solution (disease-specific factor and biopsychosocial factor), with appropriate eigenvalues. To our knowledge, this scale is the first statistically analyzed outcome scale for childhood eating disorders.

The disease-specific factor included three outcomes (EA, FF, and BD), which were drawn from the DSM-5 eating disorder criteria [15]. At the first visit assessment, the subtotal for the disease-specific factor was not significantly correlated with BMI-SDS. This may indicate that patients with early onset-type eating disorder (20% of cases were younger than age 10 years in the present study) may find it hard to describe or express their fear of being fat or body image distortion precisely at the initial intervention. Alternatively, the proportion of eating disorder types in the present study might have affected the absence of significance. About one-third of participants in this study had ARFID; these patients may show less fear of being fat and body image distortion, which might account for the low scores for the disease-specific factor. Furthermore, several psychometric studies have shown that the patient’s emotional state or interpersonal relationships were more relevant to pathophysiology in children with ARFID than in children with typical AN [39, 40].

The biopsychosocial factor included six outcomes (BW, PC, AS, FA, SA, and RF). This factor was characterized by inclusion of psychosocial assessments that mainly covered relationships with parents, friends, and school. Although the Morgan-Russell Outcome Assessment Schedule has been widely used and includes psychosocial scales, those scales are not specific to children as they cover marriage, sexual identity, and image of menstruation [12]. Given the increasing prevalence of early onset-type eating disorders, such items may not be suitable for an outcome scale for children. Both the biopsychosocial factor subtotal and the total score were significantly correlated with BMI-SDS at the first visit, indicating that biopsychosocial assessment was more significant for the pathogenesis of eating disorders in participating children. Several reports have suggested that psychosocial and socioeconomic status are strongly associated with onset or severity of childhood eating disorder, and family/social relationships and support are critical for their remission [19, 23–27]. The childhood eating disorder outcome scale placed emphasis on comprehensive psychosocial aspects; therefore, the scale may be useful for assessing therapeutic components that need more support for remission.

Furthermore, our procedure of factor analysis for the selected outcomes, with exclusion of three non-significant items (ME, RS, and RP) made the childhood scale more reliable. The RS and RP items appeared to be important for a patient’s remission; however, a more detailed definition for the categorization of these items may be necessary to exclude physician subjectivity. For example, disease recognition differed between mothers and fathers and between class teachers and school nurses. Raters also often had difficulty in deciding scores; therefore, we excluded those two scales. The exclusion of ME was based on agreement about the consideration of younger cases, and was consistent with DSM-5 criteria, from which amenorrhea was excluded [15].

In our study, 46 children had psychiatric comorbidity (e.g., autism spectrum disorder, depression, obsessive compulsive disorder), which are considered to have significant adverse effects on the prognosis for eating disorders [9, 13, 41]. The mean outcome score in children with psychiatric comorbidity was higher than that in total cases (17.0 vs. 13.3); however, the mean BMI-SDS did not differ between the two groups (− 3.2 vs. − 3.5). At the 12-month follow-up, changes in mean BMI-SDS did not differ between children with psychiatric comorbidity and total cases. This indicated that the presence of psychiatric comorbidity had little effect on body weight changes. Similarly, the presence of physical illness showed a slightly higher mean outcome score (15.1) among those with comorbidities compared with total cases; however, there was little effect on the mean BMI-SDS (− 2.9) in this group (data not shown).

As we planned to measure the childhood eating disorder outcome scale prospectively, it was consecutively measured from the initial visit to 12 months. This qualitative prospective study design was suitable to assess patient outcome and minimized research bias. The outcome scores in the majority of cases showed improvement, accompanied by recovering BMI-SDS, and the changes of total scale score from the initial visit to 12 months significantly reflected the changes in BMI-SDS. These results indicate that this outcome scale could be used for assessment of therapeutic effects during the course of treatment.

There were several limitations to this study. First, although 46 patients had psychiatric comorbidity (e.g., autism spectrum disorders, depression, obsessive compulsive disorder), involvement of these comorbidities was not included or considered as weighting elements for the outcome score. Although those comorbidities have been considered as having significant adverse effects on the prognosis for eating disorders [9, 13, 41], the degree of severity of comorbidities may differ among disorders, or among patients with each comorbid disorder. Therefore, we did not include the presence of comorbidity in the outcome scale. The presence of comorbidity could be assessed as factor predictive of prognosis in future research. Second, it may be better to distinguish ARFID and AN at the initial assessment and conduct analyses separately for rating purposes. However, initial assessment for differential diagnosis of these two types of childhood eating disorders may be difficult. We did not consider the type of eating disorder, because comparable improvements in weight and psychopathology have been reported as outcomes for patients with ARFID and those with AN [42–44]. Furthermore, physicians sometimes have difficulty making a differential diagnosis between ARFID and AN, as there are cases in which children with AN do not express fear of being fat or body image distortion. After induction of nutritional therapy, children with an eating disorder tended to present with a fear of being fat or other characteristics specific to AN. Further research is needed to develop outcome scales that specify the type of eating disorder. Third, although all initial visit data for 131 cases were included in the factor analysis, 22 of the 131 children dropped out or were referred to psychiatric care before the 12-month evaluation. Fourth, each clinician completed the outcome scale ratings for their patients without a blinded double coding, which gives a possibility of rater variability. Furthermore, types of treatment were not evaluated for each case. The reasons for dropping out and type of treatment need to be further evaluated to assess more global aspects of the outcome scale. Although this study had several methodological limitations, including lack of physically meaningful aspects, an advantage of the new rating scale in the clinical context is that it may make it possible for physicians to assess a patient’s entire condition and clinical process of disease severity.

In conclusion, we developed a childhood eating disorder outcome scale that comprised disease-specific and biopsychosocial factors. We showed that the biopsychosocial score was significantly associated with BMI-SDS and that change in the total score through therapeutic intervention reflected improvement in BMI-SDS. This scale is able to assess the severity of childhood eating disorder and identify therapeutic fragments that should receive more support, which would lead to a better prognosis for children with eating disorders.

## Data Availability

The datasets of this study are available from the corresponding author on reasonable request.
